# Undifferentiated Pancreatic Carcinoma with Osteoclast-Like Giant Cells: a Case Report

**DOI:** 10.1007/s12029-013-9572-9

**Published:** 2014-01-03

**Authors:** Noureddine Njoumi, Faricha Hassan Elalami, Gilles Attolou, Omar Saoud, Mohamed Elabsi, Mahjoub Echarrab, Mohamed Elouannani, Abdelkader Errougani, Mohamed Amraoui, Mohamed Rachid Chkoff

**Affiliations:** Department of Visceral Surgical Emergency, Ibn Sina Hospital, CHU Ibn Sina, Rabat, Morocco

## Introduction

Undifferentiated pancreatic carcinoma with osteoclast-like giant cells (UPC-OGCs) is a rare neoplasm with frequency of 0.2 % of reported pancreatic carcinomas [1]. Tumors with osteoclast-like giant cells have rarely been reported in a variety of extra-skeletal sites. On the alimentary tract, the pancreas is the most concerned [2, 3]. A number of terms have been used to describe variants of undifferentiated pancreatic carcinoma, especially pleomorphic carcinoma, pleomorphic giant cell carcinoma, sarcomatoid carcinoma, spindle cell carcinoma, anaplastic carcinoma, undifferentiated carcinoma, and osteoclastic or pleomorphic giant cell tumors [4].

Histologically, the tumor combines two main cell populations, a malignant mononuclear cells population and the scattered nonneoplastic osteoclast-like giant cells [3, 5].

Since the first description by Rosai in 1968 [6], there have been only a few cases reported in the world literature. Our aim is to describe a new case of undifferentiated carcinoma of the pancreas with osteoclast-like giant cells which was early diagnosed, with a review of literature.

## Case Report

**Fig. 1:**
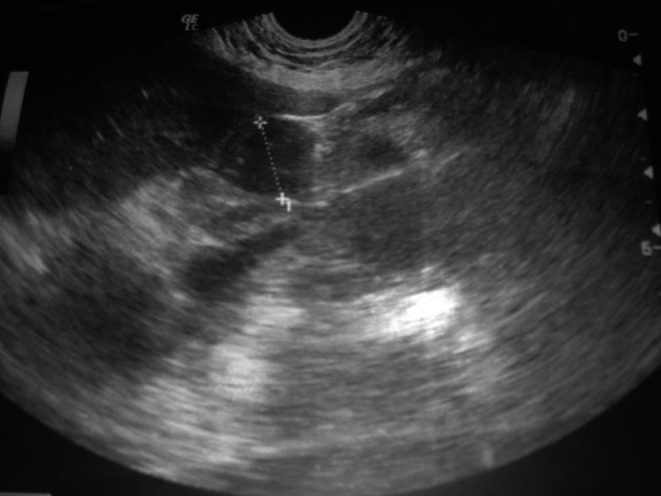
Ultrasound: hypoechogenous, nodular formation in the pancreatic body

**Fig. 2:**
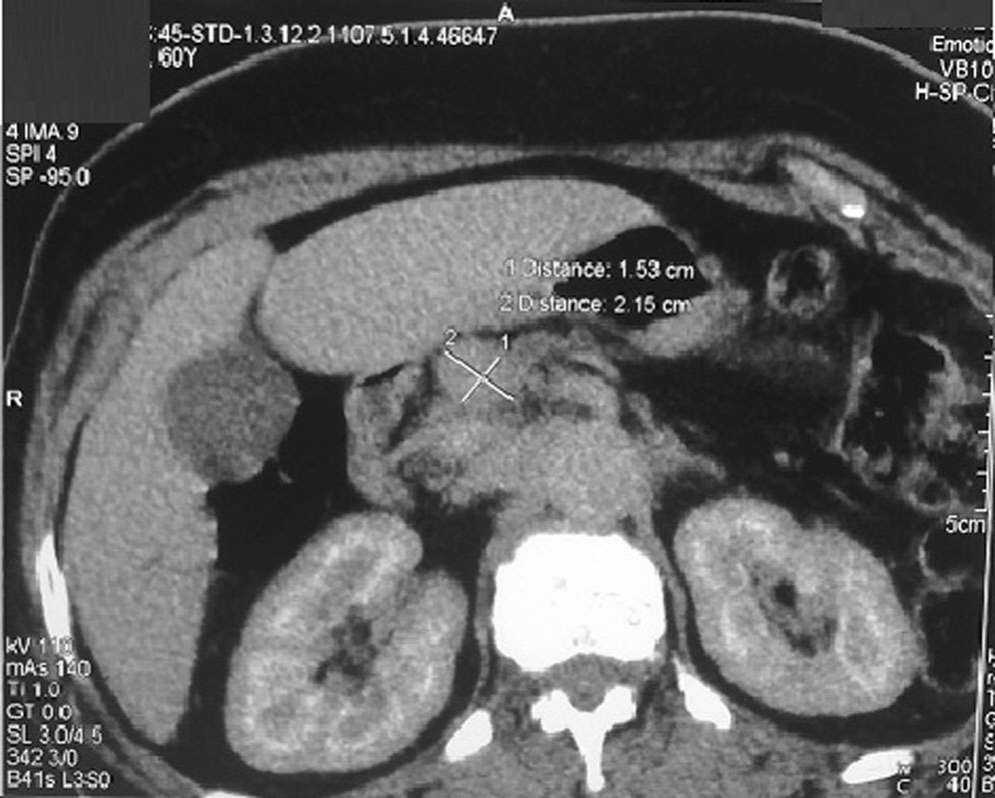
Computed tomography: limited tissue mass in the pancreatic body, measuring 2.1 × 1.5 cm

**Fig. 3:**
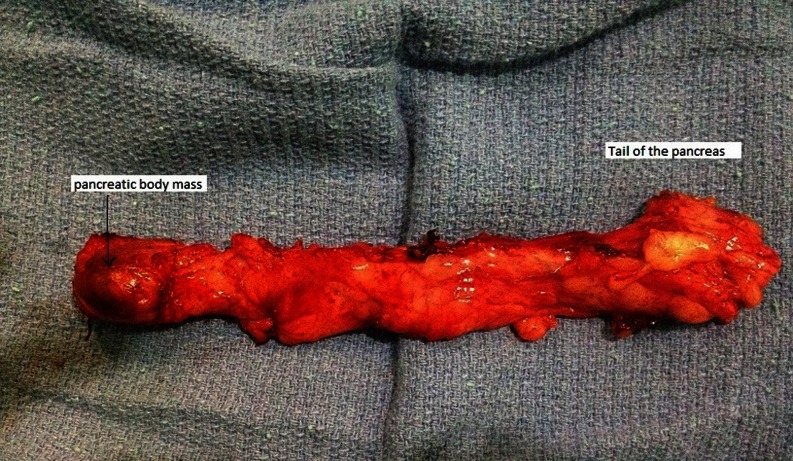
Surgical specimen: corporeo-caudal pancreatectomy

**Fig. 4:**
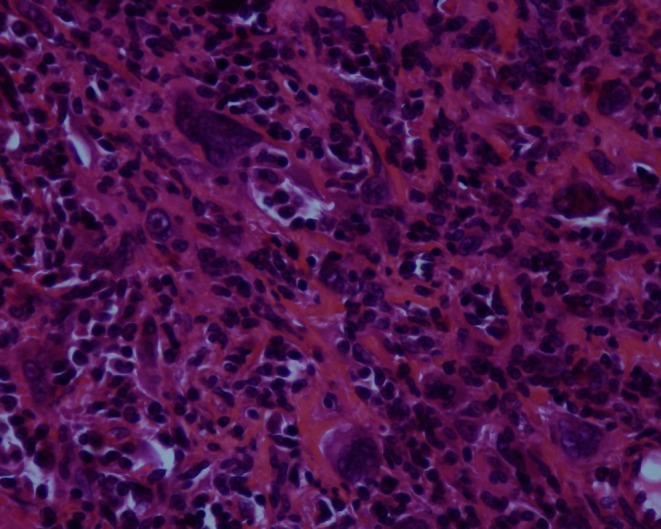
Presence of osteoclast-like giant cells (magnification of ×40)

**Fig. 5:**
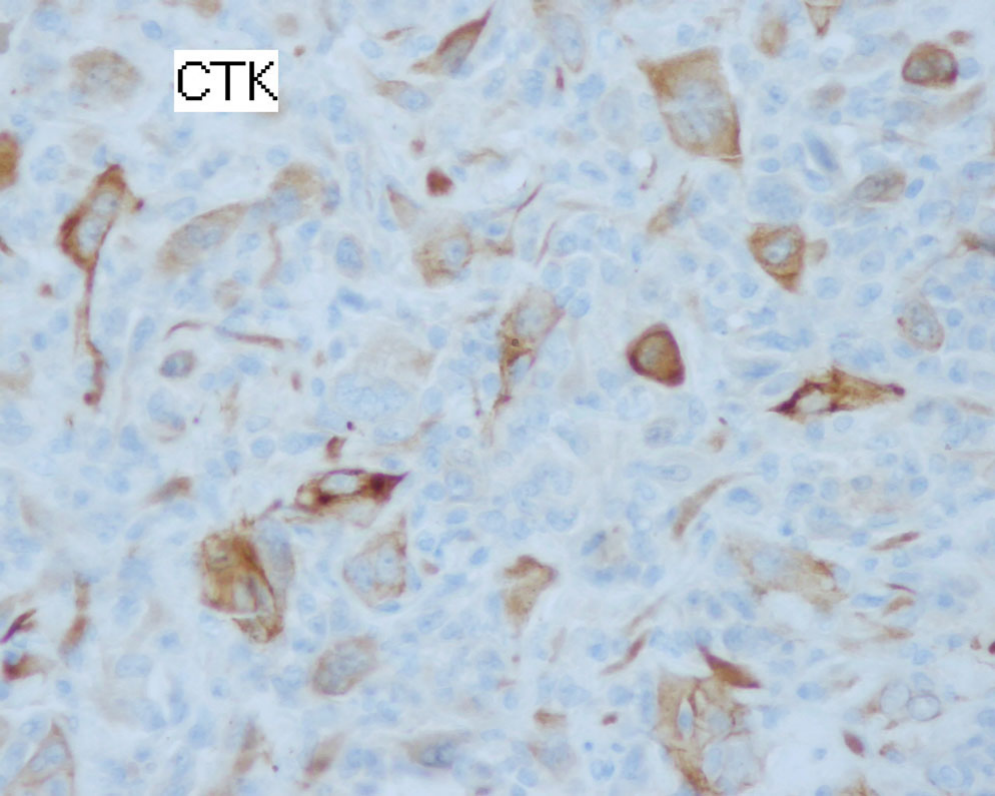
Marking of tumor cells with anti-cytokeratin antibody (magnification of ×40)

A 60-year-old woman with no medical history was referred with upper abdominal pain of five-months duration. She did not report any weight loss or fever. The physical examination and vital signs were normal. Her abdomen was soft and nontender. Serum chemistries were within the normal range except for a glucose level of 2.7 g/dL. Serum cancer antigen 19–9 was elevated to 62 UI/mL (normally lower to 37 UI/mL), and carcinoembryonic cancer antigen was normal. Ultrasound examination demonstrated a 2.4-cm well-defined, hypoechogenous, nodular formation in the pancreatic body (Fig. 1). Abdominal computed tomography revealed a very limited tissue mass in the pancreatic body, measuring 2.1 × 1.5 cm responsible for dilatation of main pancreatic duct (5 mm) with upstream pancreatic atrophy (Fig. 2). Surgical exploration shows a very limited mass in the body of the pancreas without local invasion, lymph nodes, or other metastasis. The patient received distal pancreatectomy with spleen preservation (Fig. 3). She had an uneventful postoperative recovery. Pathology showed a mixed macroscopic structure and a diffused tumor proliferation in microscopy. The tumor cells had moderate to marked cytonuclear atypia with multiple multinucleated giant cells. Atypical mitotic figures were also observed. No intravascular tumor emboli were noticed. The tumor proliferation was 2 mm from the edge of resection. The adjacent pancreatic parenchyma was the seat of chronic pancreatitis lesions. Immunohistology study demonstrated an expression of anti-cytokeratin antibodies AE1/AE3, negative staining for CD117 and PS100, and a proliferation rate of 70 %. The giant cells were mostly positive for CD68 (Figs. 4 and 5). The tumor was classified as T1N0M0; an adjuvant chemotherapy was indicated during the meeting of multidisciplinary consultation, but the patient refused until now any postoperative treatment. Nine months after surgery, the patient is still alive and had no recurrence.

## Discussion

Undifferentiated pancreatic carcinoma with osteoclast-like giant cells is a rare malignancy, which accounts for 2–7 % of all pancreatic cancers [4, 7]. There are three major subtypes of undifferentiated pancreatic carcinoma that include giant cell carcinoma, spindle cell carcinoma, and round cell carcinoma [4, 8]. The giant cell carcinoma of the pancreas presents three subtypes: osteoclastic, pleomorphic, and mixed [1, 9]. The first one like in our case is estimated at 0.2 % of the total number of pancreatic carcinomas [1]. It was initially reported in the pancreas by Juan Rosai in 1968 [6]. According to the literature and similar to pancreatic adenocarcinoma, giant cell tumors of the pancreas tend to occur in the elderly [1, 4, 9]. The average age of patients is around 60 years, ranging from 28 to 88 years. Males and females appear to be affected in a fairly equal ratio [3, 10, 11].

Clinically, the reported cases do not show peculiarity compared to conventional pancreatic ductal adenocarcinoma [11–13]. The main symptoms are abdominal pain and weight loss. Jaundice occurs if the tumor is located in the head of the pancreas. A palpable mass may be found in cases of great tumors. Invasion into adjacent structures is frequent. Nodal or intra-abdominal metastasis is found in approximately 50 % of patients at the time of diagnosis [2, 11].

Radiological investigations often show a mixed cystic and solid tumor with an average size around 6 cm. Most tumors arise in the head or body of the pancreas [3, 12].

The differential diagnosis of UPC-OGCs includes cystic lesions like mucinous cystadenomas or cystadenocarcinomas, pancreatic pseudocysts, pseudopapillary tumors, and also solid pancreatic tumors like ductal pancreatic carcinomas. Solid tumors may be homogenous on computed tomographic imaging; however, they can also be very inhomogeneous, as focal hemorrhage or necrosis is frequently found [3, 12, 13].

At the time of diagnosis, more than 80 % of tumors are already larger than 5 cm and 50 % even larger than 10 cm. In more than 50 cases, in which tumor size was documented, only three neoplasias measured less than 3 cm [3]. The tumor size in our case is 2.5 cm, and this shows the early character of diagnosis and treatment motivated by chronic pancreatitis symptoms.

Histologically, UPC-OGCs are composed of two distinct cell types: OGCs and mononuclear cells. Mononuclear cells originating from epithelium constitute the tumor itself, while OGCs appear to be a paraneoplastic product of this rare malignant tumor [2].

More recent molecular studies have demonstrated the presence of K-ras mutations in the mononuclear cells, suggesting that they comprise the neoplastic component of UPC-OGCs and that they may arise from intraductal epithelial precursors [5]. The frequency of K-ras mutation appears to be higher in pancreatic carcinoma. More than 93 % of these K-ras mutations are located in codon 12, demonstrating the very high specificity of K-ras gene mutation for pancreatic carcinoma. These findings suggest that the K-ras oncogene plays a critical role in the oncogenesis of UPC-OGCs [2, 12].

As reported in our patient, tumor cells possess cell surface markers and a histologic appearance consistent with an epithelial cell type [14].

Concerning the treatment, it is recommended to follow protocols established for ductal pancreatic adenocarcinoma. In fact, only complete tumor resection may prolong survival [1, 12–14].

A clear understanding of the clinical behavior of UPC-OGCs is limited, given the small number of reported cases. The overall prognosis for these tumors seems to be slightly more favorable than for pancreatic ductal adenocarcinoma. A literature review by Shiozawa et al. consisting of 32 cases of UPC-OGCs, found the mean survival postdiagnosis or postsurgical resection to be 20.4 months [4, 5, 15].

In conclusion, undifferentiated pancreatic carcinoma with osteoclast-like giant cells is a rare entity described in a few case reports. These tumors tend to be large, with a heterogeneous echotexture. The diagnosis is generally made on surgical specimen, and the prognosis is poor.
